# Defining a Synovial Fluid White Blood Cell Count Threshold to Predict Periprosthetic Infection after Shoulder Arthroplasty

**DOI:** 10.3390/jcm11010050

**Published:** 2021-12-23

**Authors:** Laura Elisa Streck, Chiara Gaal, Johannes Forster, Christian Konrads, Sebastian Philipp von Hertzberg-Boelch, Kilian Rueckl

**Affiliations:** 1 Department of Orthopaedic Surgery, Koenig-Ludwig-Haus, University of Wuerzburg, 97070 Wuerzburg, Germany; laura.e.streck@gmail.com (L.E.S.); c-gaal.klh@uni-wuerzburg.de (C.G.); s-boelch.klh@uni-wuerzburg.de (S.P.v.H.-B.); 2 Institute for Hygiene and Microbiology, University of Wuerzburg, 97070 Wuerzburg, Germany; forster_j1@ukw.de; 3 BG Trauma Center, Department for Traumatology and Reconstructive Surgery, University of Tübingen, 72076 Tubingen, Germany; christian.konrads@gmail.com

**Keywords:** upper extremity, joint infection, joint aspiration, leucocyte count, cutibacteria, ICM, MSIS, IDSA, WBC

## Abstract

Background: The diagnosis of periprosthetic shoulder infection (PSI) requires a thorough diagnostic workup. Synovial fluid aspiration has been proven to be a reliable tool in the diagnosis of joint infections of the lower extremity, but shoulder specific data is limited. This study defines a threshold for synovial fluid white blood cell count (WBC) and assesses the reliability of microbiological cultures. Methods: Retrospective study of preoperative and intraoperative fluid aspiration of 31 patients who underwent a revision of a shoulder arthroplasty (15 with PSI defined by IDSA criteria, 16 without infection). The threshold for WBC was calculated by ROC/AUC analysis. Results: WBC was significantly higher in patients with PSI than in other patients. A threshold of 2800 leucocytes/mm^3^ showed a sensitivity of 87% and a specificity of 88% (AUROC 0.92). Microbiological cultures showed a sensitivity of 76% and a specificity of 100%. Conclusions: A threshold of 2800 leucocytes/mm^3^ in synovial fluid can be recommended to predict PSI. Microbiological culture has an excellent specificity and allows for targeted antibiotic therapy. Joint aspiration presents an important pillar to diagnose PSI.

## 1. Introduction

Shoulder arthroplasty has evolved to become a successful treatment not just in cuff-tear arthroplasties. However, complications are reported in 10% of the patients following anatomic total shoulder arthroplasty (TSA) and in 16% following reverse shoulder arthroplasty (RSA) [1]. Hereof, prosthetic shoulder infection (PSI) is one of the most harmful. It occurs in 0.5–6% of primary and in up to 15% of revision arthroplasties [1,2,3,4]. Yet varying clinical symptoms require a thorough diagnostic workup to decide about momentous treatment options when facing possible PSI. Analysis from synovial fluid aspiration is an established pillar of this workup. It can provide synovial fluid white blood cell count (WBC), alpha-defensin- and leucocyte-esterase-levels, as well as microbiological cultures [4,5].

Synovial WBC has been proven to be a reliable tool in the diagnosis of infections of the lower extremity and various studies provide corresponding data. In contrast, shoulder specific data are limited to very few studies with small case numbers [6]. The transfer of thresholds established in the lower extremity is debatable and there is no consensus about joint specific cut-off values for the shoulder [7,8,9].

The reported sensitivity of microbiological cultures from preoperative joint aspiration (PA) varies widely between 9–85% and its value is under debate [4,10,11,12,13]. The shoulder specific bacterial spectrum, characterized by a majority of cutibacteria and coagulase-negative staphylococci (CoNS), complicates the assessment of microbiological cultures and is likely to increase the risk of false negative results [3,4,12].

The current study aims to address the following research questions: What is an appropriate threshold for synovial WBC in the diagnosis of PSI? How reliable are microbiological cultures from shoulder joint aspiration?

## 2. Materials and Methods

The study retrospectively investigated results from shoulder joint aspiration (preoperative aspiration, PA) prior to revision surgery of a shoulder prosthesis of 31 consecutive patients in a single center between 2005 and 2020.

C-reactive protein (CRP), erythrocyte sedimentation rate (ESR) and WBC from peripheral blood were determined preoperatively.

All patients underwent diagnostic shoulder joint aspiration prior to surgery. None received antibiotic therapy prior to PA. PA was performed with a sterile canula from either an anterior or dorsal approach after 3 min of disinfection by alcoholic skin disinfectant (Octeniderm^®^, Schuelke and May, Norderstedt, Germany). Aspirated synovial fluid was transferred to a sterile test tube to calculate the synovial fluid white blood cell count (WBC). In 29 cases, blood culture bottles were inoculated for aerobic (BacT/ALERT^®^ FA Plus) and anaerobic (BacT/ALERT^®^ FN Plus) cultures (bioMérieux, Marvie-L’Étoile, France). Blood culture vials were incubated for 14 days or until they flagged positive in the BacT/ALERT^®^ 3D System (bioMérieux, Marvie-L’Étoile, France). Species diagnosis was established by Matrix-assisted laser desorption/ionization—time of flight (MALDI-TOF) from solid culture media.

Intraoperative aspiration and biopsies from revision surgery were taken right after opening the joint capsule and before starting perioperative intravenous antibiotic prophylaxis or therapy. At least three biopsies were taken from membranes, resected bone and directly surrounding soft tissue. Sterile sample vessels were opened under laminar flow and fractioned. Cultures were nourished in brain–heart infusion bouillon and Thioglycolate bouillon and plated out on Candida chrome agar, Columbia blood agar, cooking blood agar, MacConkey agar and Schaedler agar. Cultures were incubated for 14 days. Species were differentiated throughout mass spectrometry (Vitek MS, bioMérieux, Marvie-L’Étoile, France) followed by testing of sensitivity (Vitek 2, bioMérieux, Marvie-L’Étoile, France). WBC was calculated using a Neubauer counting chamber (Diagonal GmbH & Co-KG, Muenster, Germany).

We retrospectively divided the patients into a prosthetic shoulder infection (PSI) group and a non-infection group. The diagnosis of infection was based on the Infectious Diseases Society of America (IDSA) criteria [14]. A total of 15 patients met the criteria (PSI group), while 16 were grouped in the non-infection group. Demographic data is provided in Table 1.

Statistical tests were performed using SPSS Statistics^®^ Version 24.0 (IBM, New York, NY, USA) and Microsoft Excel^®^ Version 1908 (Microsoft, Washington, DC, USA). Descriptive statistics were performed to describe means, range and standard deviation (SD). Normal distribution was tested with Shapiro–Wilk test and a Levene’s test was used to test for homogeneity of variances. Student-t test (for metric and normally distributed variables; blood WBC) or the Mann-Whitney U test (for not normally distributed variables; CRP, ESR, WBC) were performed to identify significance of the differences. The significance level was set at α < 0.050. The presence of equal or unequal variances was determined using the Levene’s test. Cross tables were used to calculate sensitivities and specificities. Correlations between synovial WBC and blood infections markers were tested with bivariate correlation analysis. A receiver operating characteristic (ROC) was plotted for synovial WBC depending on the diagnosis of PSI to depict the correlation between sensitivity and specificity. Area under curve (AUC) analysis, Youden’s index and positive likelihood ratio (LR) were calculated to quantify the quality of the test.

The study was approved by the ethic committee of the author’s institution (235/16-mk). Informed consent was obtained from all subjects involved in the study.

## 3. Results

### 3.1. Blood Infection Markers

Table 2 depicts the preoperative results of c-reactive protein (CRP), erythrocyte sedimentation rate (ESR) and WBC from peripheral blood. There was a significant difference between the PSI group and the non-infection group for CRP (*p* = 0.004) and ESR (*p* = 0.002). Both markers also showed positive correlations with synovial WBC (*p* < 0.001).

### 3.2. Microbiological Cultures

The non-infection group showed no positive microbiological cultures, neither from PA nor intraoperative. Amongst the negative intraoperative cultures was one case that showed intraoperative growth of *C. acnes* in only 1 of 3 specimens and was therefore considered as a contamination.

Two out of 15 samples in the PSI group did not provide enough fluid for microbiological culture in PA. These cases shielded growth of *C. avidum*, respectively, *S. aureus* in intraoperative samples.

**Figure 1:**
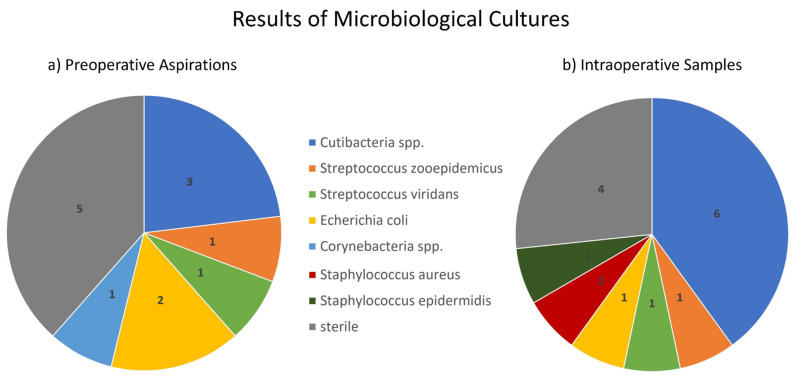
Results of microbiological cultures from (**a**) preoperative aspiration and (**b**) intraoperative samples. Numbers in the diagram represent case numbers. The percentage of sterile samples decreased from 38% in PA to 27% in intraoperative cultures. The percentage of cutibacteria species (spp.) increased from 23% in PA to 40% in intraoperative cultures.

Thus, microbiological cultures are available from 13 out of 15 PSI patients. Additionally, 8 out of 13 cultures from PA and 11 out of 15 intraoperative cultures were positive. Detailed information on the bacterial spectrum is provided in Figure 1. It was also found that 23% of the PA samples yielded growth of cutibacteria, whereas the rate was 40% in intraoperative samples. There was a shift from sterile samples in PA to *C. acnes* and *S. epidermidis* in one case, respectively.

The sensitivity of microbiological cultures from PA was 76%, and the specificity was 100%. If referenced to the IDSA criteria, the sensitivity of intraoperative microbiological cultures was 73%, and the specificity was 100%. According to the definition of PSI, based on IDSA criteria and with reference to intraoperative samples, the rate of culture negative infections was 26%.

### 3.3. Synovial WBC in Preoperative Shoulder Joint Aspiration

**Figure 2:**
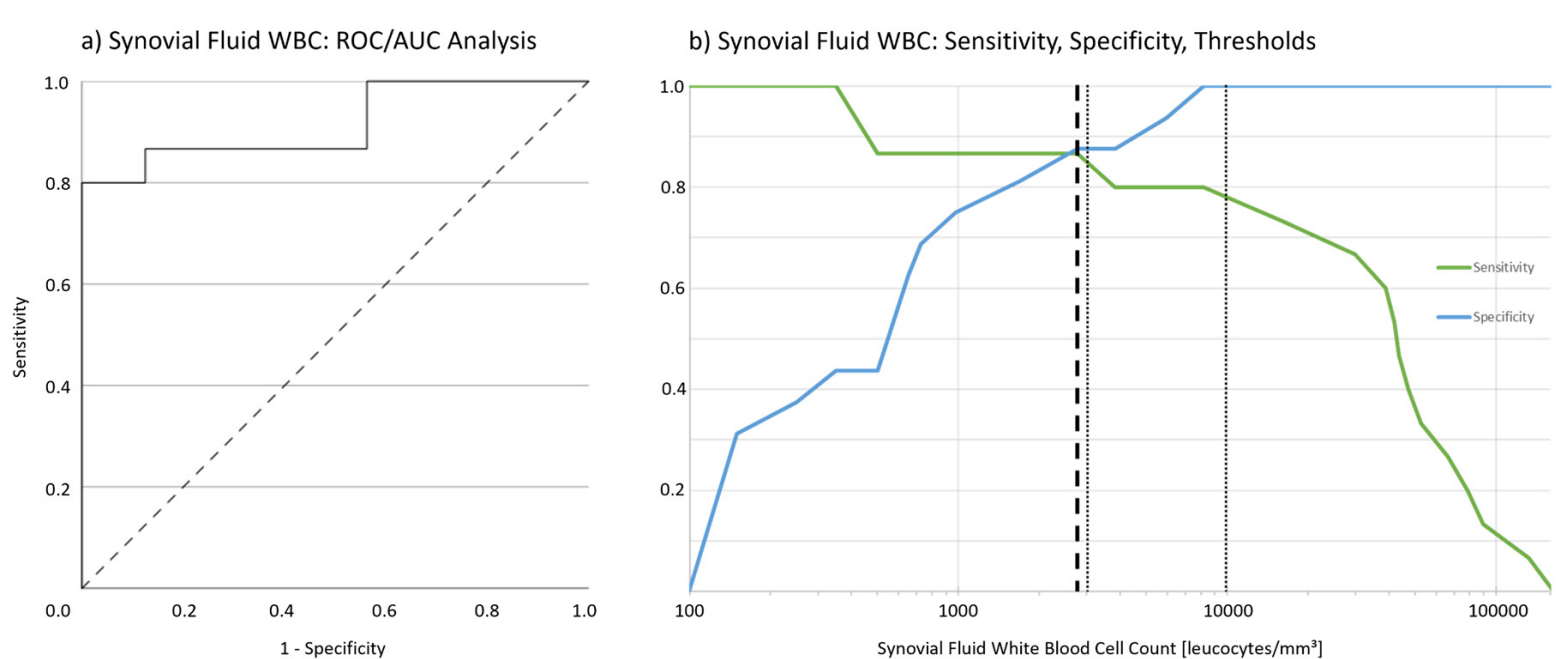
(**a**) ROC/AUC analysis for the sensitivity and specificity of synovial white blood cell count (WBC) in PSI. 2800 leucocytes/mm^3^ showed a sensitivity of 87% and a specificity of 88% (AUROC 0.92, Youden index 0.74). (**b**) Sensitivity and specificity of synovial white blood cell count in the diagnosis of PSI (according to IDSA criteria). WBC was plotted in decadic logarithmic scaling. The dashed vertical line represents the calculated threshold for PSI (2800 leucocytes/mm^3^). Dotted vertical lines represent WBC thresholds for chronic (3000 leucocytes/mm^3^) and acute (10,000 leucocytes/mm^3^) periprosthetic hip and knee infections according to ICM criteria.

Mean synovial WBC in patients with PSI was 48,600 leucocytes/mm^3^ (range 400–165,000, SD 44,000), mean synovial WBC in non-infection patients was 1200 leucocytes/mm^3^ (range 100–7600, SD 2000), the difference was significant (*p* = 0.001). A threshold of 2800 leucocytes/mm^3^ showed a sensitivity of 87% and a specificity of 88% in the ROC analysis (AUROC 0.92, Youden index 0.74, Figure 2).

Sensitivity, specificity and likelihood ratios (LR) of different WBC thresholds are presented in Table 3.

A subgroup of patients (*n* = 4) was: PA (a) had negative cultures or (b) did not provide enough fluid for cultures, but showed positive intraoperative cultures, and had an average WBC of 56,400 leucocytes/mm^3^ (range 400–165,000, SD 64,100). It did not differ (*p* = 0.186) from patients with both positive cultures from PA and intraoperatively (*n* = 7, 63,100 leucocytes/mm^3^, range 41,500–100,000, SD 21,000). There was no significant (*p* = 0.278) difference in WBC between patients with intraoperative detection of cutibacteria (*n* = 6, 73,900 leucocytes/mm^3^, range 400–165,000, SD 50,900) or other bacteria (*n* = 5, 44,800 leucocytes/mm^3^, range 41,500–80,000, SD 18,900).

## 4. Discussion

WBC from PA can be obtained quickly and easily. Defining a consensus for a shoulder specific threshold value for WBC in PA to predict periprosthetic joint infection is essential for the diagnostic workup in daily practice. The current study revealed a threshold value of 2800 leucocytes/mm^3^ to predict PSI with a good sensitivity of 87% and a specificity of 88%. To the author’s best knowledge, this value is based on the biggest patient cohort investigated to date. Few studies have reported on WBC from PA of the shoulder and presented inhomogeneous results. Even a current literature review on the diagnosis of PSI by Jauregui et al., published 2021, did not report on WBC [5]. Strahm et al. recommended a cut-off of 12,200 leucocytes/mm^3^ for WBC in PSI. The calculation was based on 13 shoulders with PSI and 6 non-infection shoulders [8]. In our results, a higher threshold (i.e., 8200 leucocytes/mm^3^) would have increased the specificity (100%), LR and Youden index but the simultaneous drop of sensitivity would significantly increase the risk of missing a true infection. Another study by Nodzo et al., reported on the WBC in patients with periprosthetic *C. acnes* infections. The mean WBC for patients with PSI was 750 leucocytes/mm^3^ and significantly lower than in knee infections [7]. Based on this study, the 2018′s International Consensus Meeting on Periprosthetic Joint Infections in Philadelphia (ICM Philly) recommended orientation on low grade hip infections and therefore defined a cut-off of 3000 leucocytes/mm^3^ for PSI [15]. This value is approximately supported by the results of the present study.

Due to the retrospective design and small case number, this study did not distinguish between acute and chronic PSI. The threshold for PSI was slightly lower than the cut-off for chronic periprosthetic infection of the hip and knee (3000 leucocytes/mm^3^), and far below the threshold for acute infection (10,000 leucocytes/mm^3^) recommended by the ICM Philly [16]. This supports the suggestion that PSI is likely to come along with lower WBC than infections of the lower extremity. Thus, PSI are prone to be missed if lower extremity thresholds were uncritically and directly adopted to the shoulder.

The subgroup analysis revealed no significant differences in WBC between patients with cutibacteria, compared to other bacteria; nor between patients with negative PA and positive intraoperative cultures compared to positive PA.

In the current study, microbiological culture from PA had a sensitivity of 76% to detect PSI. One reason for the limited sensitivity might be the shoulder specific bacterial spectrum, characterized by a majority of cutibacteria and CoNS [11,17,18]. These pathogens are typically low-virulent and biofilm-forming, which is likely to negatively affect the results of PA [3,12,19,20,21]. Even while being present as sessile pathogens within a biofilm in a tissue, especially cutibacteria and CoNS, might not be detectable in an aspirate in a planktonic state [19]. Furthermore, cutibacteria are anaerobe bacteria and prone to be missed by culture due to inadequate culture media or long transport time [20]. The adequate period of incubation is controversially discussed. Frangiamore et al. considered true positive cultures to be positive within 4–6 days and mentioned the risk of false positive results in prolonged incubation [22]. Pottinger et al. found that only 86% of Cutibacterium acnes cultures were positive within 14 days [21]. The current study is based on an incubation period of 14 days, which is in accordance with the current literature [7]. The hypothesis that low-virulent organisms impair the sensitivity of PA, is supported in the current study as the percentage of cutibacteria detected in PA was only 23% compared to 40% in the intraoperative samples. Furthermore, the current cases with culture negative PA, but positive intraoperative samples, shielded growth of cutibacteria and S. epidermidis.

The relevant rate of false negative results for microbiological cultures from PA as described in literature was further confirmed in the current study [6,23,24]. This disqualifies microbiological cultures from PA to rule out PSI. However, the specificity of 100% found in this study, justifies targeted antibiotic therapy and underlines the legitimate role of PA as an important diagnostic pillar in PSI. Furthermore, PA offers not only WBC and microbiological cultures but further diagnostic tools such as alpha-defensin or leucocyte-esterase [5]. Results from PA are an essential part of common joint-infection criteria as published by the MusculoSkeletal Infection Society (MSIS) or ICM Philly [15,25].

A side aspect of this study was the assessment of intraoperative microbiological cultures. Compared to cultures from PA, intraoperative cultures showed a superior detection rate for bacteria in general and a higher rate of cutibacteria in particular. Nevertheless, the rate of culture negative infections was 26%. This is even higher than the rates reported for periprosthetic hip and knee infections of 5–25% [26].

Widely established diagnostic criteria such as IDSA or MSIS criteria that were developed for hip and knee infections are essentially based on microbiological cultures (from PA and intraoperative biopsies) [14,25]. Low detection rates, concerning both PA and intraoperative cultures, might therefore increase the risk of missed PSI if applying preexisting criteria from the lower extremity to the shoulder. While average CRP and ESR were significantly higher in patients with PSI compared to others, there were some patients with PSI whose CRP and ESR were within the normal range. The individual variability makes it crucial to include all available parameters, and demands tailored diagnostic scores. Consequently, the first diagnostic criteria to accommodate the shoulder specific situation have been presented by the ICM Philly in 2018 [15]. Further studies and more data are necessary to further evaluate this score and generally increase the shoulder specific evidence to allow for a profound assessment of diagnostic parameters.

The following limitations to the current study must be considered: (1) The study design was retrospective. (2) The percentages of PMN, alpha-defensin, and leukocyte esterase levels are not available. (3) The limited number of patients. However, to the authors’ best knowledge, it is the largest cohort to date. (4) There is some evidence that using EDTA tubes to prevent clotting of synovial fluid might improve WBC calculation and that automated counting might be superior to manual counting [27] (5) PSI was defined by IDSA criteria. This definition has shortcomings. However, beside MSIS and ICM criteria, IDSA is the only score that does not include WBC count. Therefore, it is the best score to assess the current research questions.

## 5. Conclusions

To the authors best knowledge, the current study provides the largest study cohort on WBC into periprosthetic joint infection of the shoulder to date. The current results propose a threshold of 2800 leucocytes/mm^3^ in synovial fluid from PA that provided a sensitivity of 87% and a specificity of 88% to predict PSI.

The sensitivity of microbiological cultures from PA was only 76%; in particular, cutibacteria and CoNS are prone to be missed. By itself, neither results from PA nor from intraoperative cultures are valid enough to diagnose or rule out PSI with certainty. However, PA culture has an excellent specificity and allows for targeted antibiotic therapy. In daily practice, PA is easily available in a short amount of time. It presents an important pillar to diagnose PSI and its inclusion in diagnostic scores is justified.

## Figures and Tables

**Table 1:** Demographic data of the study population.

Diagnosis	Number of Patients	Age	Sex
PSI	15	72 years (range 55–84)	7 male 8 female
Non-infection	Periprosthetic fracture: 2 Instability: 3 Aseptic component loosening: 5 Shoulder dislocation: 2 Rotator cuff insufficiency/superior escape: 4	70 years (range 45–90)	3 male 13 female

PSI was defined according to the IDSA criteria.

**Table 2:** Blood infection markers.

Blood Infection Marker		PSI		Non-Infections		*t*-Test/U-Test	Correlation with Synovial WBC	
	normal range	mean	SD	mean	SD	*p*	Pearson correlation	*p*
CRP (mg/dl)	<0.8	5.3	4.7	1.0	1.1	0.004 *	0.686	<0.001 *
ESR (mm/h)	<25	40	21	16	12	0.002 *	0.792	<0.001 *
Blood WBC (leucocytes/mm^3^)	4000–10,000	9900	3300	8600	3000	0.237	0.349	0.054

Blood infections markers in 15 patients with PSI and 16 non-infection patients. * CRP and ESR were significantly higher in patients with PSI. Both showed correlations with synovial WBC. No significant correlation or difference was found in blood WBC.

**Table 3:** Sensitivity, specificity, Youden index and positive likelihood ratio for different WBC thresholds.

**WBC** **(Leucocytes/mm^3^)**	99	200	400	700	1000	1700	2800	3800	5900	8200	16,100	30,000	47,200	66,500	90,000	132,500
**Sensitivity**	1	1	1	0.867	0.867	0.867	0.867	0.800	0.800	0.800	0.733	0.667	0.400	0.267	0.133	0.067
**Specificity**	0	0.312	0.437	0.687	0.750	0.812	0.875	0.875	0.937	1	1	1	1	1	1	1
**Likelihood-Ratio**	1	1.5	1.8	2.8	3.5	4.6	6.9	6.4	12.7		n/a	n/a	n/a	n/a	n/a	n/a
**Youden-Index**	0	0.312	0.437	0.554	0.617	0.679	0.742	0.675	0.737	0.800	0.733	0.667	0.400	0.267	0.133	0.067

## Data Availability

The data presented in this study are available on request from the corresponding author. The data are not publicly available due to ethical restrictions.

## Abbreviations

CRP	c-reactive protein
ESR	erythrocyte sedimentation rate
ICM Philly	2018s International consensus meeting on periprosthetic joint infections in Philadelphia
IDSA	Infectious Diseases Society of America
LR	Likelihood-Ratio
MSIS	MusculoSkeletal Infection Society
PA	preoperative joint aspiration
PSI	prosthetic shoulder infection
ROC	receiver operating curve
SD	standard deviation
WBC	white blood cell count
